# Association of Elevated High Sensitivity Cardiac Troponin T(hs-cTnT) Levels with Hemorrhagic Transformation and 3-Month Mortality in Acute Ischemic Stroke Patients with Rheumatic Heart Disease in China

**DOI:** 10.1371/journal.pone.0148444

**Published:** 2016-02-05

**Authors:** Junfeng Liu, Deren Wang, Yao Xiong, Bian Liu, Zilong Hao, Wendan Tao, Ming Liu

**Affiliations:** Stroke Clinical Research Unit, Department of Neurology, West China Hospital, Sichuan University, Chengdu, P.R. China; University of Florida, UNITED STATES

## Abstract

**Background and Objective:**

Elevated levels of high sensitivity cardiac troponin T (hs-cTnT) occur in a substantial proportion of patients with acute ischemic stroke (AIS) and can predict poor outcome and mortality after stroke. Whether elevated hs-cTnT levels can also predict hemorrhagic transformation (HT) or prognosis in AIS patients with rheumatic heart disease (RHD) remains unclear.

**Methods:**

Data from the Chengdu Stroke Registry on consecutive AIS patients with RHD admitted to West China Hospital within1 month of stroke onset from October 2011 to February 2014 were examined. Clinico-demographic characteristics, HT, functional outcomes and stroke recurrence were compared between patients with elevated hs-cTnT levels(≥14ng/L) and patients with normal hs-cTnT levels (<14ng/L).

**Results:**

The final analysis involved 84 patients (31 males; mean age, 61.6±12.2years), of whom serum hs-cTnT levels were elevated in 58.3%. Renal impairment was independently associated with elevated hs-cTnT levels (OR 4.184, 95%CI 1.17 to 15.01, *P* = 0.028), and patients with elevated hs-cTnT levels were at significantly higher risk of HT, 3-month mortality and 3-month disability/mortality (all *P*≤0.029). After controlling for age, sex, hypertension, renal impairment and National Institutes of Health Stroke Scale score on admission, the risk of HT and 3-month mortality was, respectively, 4.0- and 5.5-fold higher in patients with elevated hs-cTnT levels than in patients with normal hs-cTnT levels.

**Conclusion:**

Elevated hs-cTnT levels are independently associated with HT and 3-month mortality in AIS patients with RHD. These results with a small cohort should be verified and extended in large studies.

## Introduction

Rheumatic heart disease (RHD) affects 1.86 per 1000 adults in China, a prevalence 10-fold higher than in the industrialized countries of Canada (0.22 per 1000) and Japan (0.14 per 1000)[1]. We have previously shown that RHD-associated stroke is common in China, and that it occurs in younger people and causes more severe symptoms at hospital admission than other types of stroke[2].That study also showed RHD to be associated with greater risk of death and stroke recurrence[2]. These results establish the importance of RHD as a cause of stroke, particularly in China, and highlight the need to identify independent predictors of prognosis in these patients at particular risk of poor outcome and complications after stroke, such as hemorrhagic transformation (HT).

Several lines of evidence point to elevated levels of cardiac troponin T (cTnT) as a prognostic indicator in stroke patients. Elevated cTnT levels occur in 5–36% of patients with acute ischemic stroke (AIS)[3–12], and they are associated with greater stroke severity on admission and higher risk of insular lobe damage, poor clinical outcome and mortality[7,11,13]. Highly sensitive (hs) cTnT assays, for which the measurements are termed hs-cTnT, have greatly expanded the potential use of cTnT levels as markers in myocardial infarction(MI), even other cardiac diseases such as structural heart disease and atrial fibrillation[14, 15] end-stage renal disease,[16]pulmonary embolism[17],and other conditions[18,19]. This literature has established hs-cTnT levels as a specific and sensitive biochemical marker of myocardial damage, and potentially of other types of tissue damage, such as stroke.

It remains unclear whether hs-cTnT levels change after stroke in patients with cardiac disease, especially RHD, as well as whether such changes predict HT or other aspects of prognosis. Therefore, the current study examined possible relationships of hs-cTnT levels with risk of HT and with overall prognosis of AIS patients with RHD in China.

## Methods

This study involved retrospective analysis of data that had been entered prospectively into the Chengdu Stroke Registry, a detailed database containing information on consecutive patients treated for stroke and stroke-related conditions at West China Hospital since January 2002[20,21]. The present study was sponsored by the National Natural Science Foundation of China and approved by the biomedical ethics committee of West China Hospital. Written informed consent was obtained from participants or their guardians.

The present study extracted data from the Registry covering AIS patients(first or recurrent)diagnosed with RHD and admitted within 1 month after stroke onset to the Department of Neurology at West China Hospital between October 2011 and February 2014.Ischemic stroke was diagnosed according to World Health Organization criteria[22], and confirmed by computed tomography or magnetic resonance imaging. RHD was diagnosed according to criteria in the International Classification of Diseases (10th edition) and confirmed by echocardiography[23]. HT was defined as hemorrhage in the infarct zone not detected by computed tomography of the head immediately after stroke, but observed later during magnetic resonance imaging[21]. Patients were excluded from our study if they refused to undergo electrocardiography or hs-cTnT analysis, if hs-cTnT levels were not obtained within 48 h after admission, or if patients did not undergo follow-up magnetic resonance imaging.

A standardized form was used to collect the following patient data: demographic characteristics, stroke severity on admission, risk factors (hypertension, diabetes mellitus, hyperlipidemia, history of stroke, current smoking and alcohol consumption), renal impairment [diagnosed based on medical history or an estimated glomerular filtration rate (eGFR) <60 ml/min/1.73 m][2],and cardiac diseases, including history of coronary artery disease and congestive heart failure. Stroke severity on admission was assessed using the National Institutes of Health stroke scale (NIHSS)[24] score.

Only hs-cTnT values measured within 48 h after admission were considered in this study, and these values were not collected for all patients, since measurements were performed at the discretion of the physician and only with the consent of the patient or guardian[25]. Based on these 48-h values, patients were classified into a group with elevated hs-cTnT levels (≥14 ng/L) and a group with normal hs-cTnT levels (<14 ng/L). The cut-off value of 14 ng/L was based on the upper limit of the 99th percentile in a normal reference population reported by the manufacturer of the hs-cTnT assay system (BioMerieux, France).

Prognosis was assessed in terms of death, disability, death/disability, and stroke recurrence at 3, 6, and 12 months after stroke. Disability was defined as a score>2 on the modified Rankin Scale (mRS)[26].

Results are reported as mean ± standard deviation (SD) for continuous variables, and as frequencies and percentages for categorical variables. In univariate analyses to examine possible associations of hs-cTnT levels with HT or prognosis, differences in categorical variables were compared between patients with elevated or normal hs-cTnT levels using the chi-squared test; differences in continuous variables were compared using Student’s t-test or the Mann–Whitney test. Variables associated with *P*< 0.10 in univariate analyses and variables previously reported in the literature to be associated with HT or prognosis were entered into a backward stepwise multivariate regression model. Two-sided *P* < 0.05 was defined as the threshold of significance. All statistical analyses were performed using SPSS 20.0 (IBM, Chicago, IL, USA).

## Results

### Baseline characteristics

During the study period from October 2011 to February 2014, 203 AIS patients with RHD were registered in the Chengdu Stroke Registry, but hs-cTnT values were measured within 48 h after admission for only 84 (41.4%). The characteristics of these 84 patients did not differ significantly in baseline characteristics from the other 119 who were excluded from our study (Table 1).

**Table 1:** Baseline characteristics of Chinese acute ischemic stroke patients with Rheumatic Heart Disease from the Chengdu Stroke Registry who were included or excluded from the present study.

Variable	Included (*n* = 84)	Excluded (*n* = 119)	*P*	OR (95%CI)
**Age,yr**	61.58(12.24)	58.79(12.7)	0.119	
**Female**	53(63.1)	78(65.5)	0.951	1.019(0.568–1.826)
**Time to admission, h**	70.11(123.34)	70.24(91.53)	0.993	
**Risk factors**				
**Hypertension**	20(23.8)	23(19.3)	0.487	1.304(0.662–2.568)
**Diabetes mellitus**	2(2.4)	8(6.7)	0.201	0.338(0.070–1.636)
**Hyperlipidemia**	2(2.4)	3(2.5)	0.949	0.943(0.154–5.771)
**Seizure**	3(3.6)	1(0.8)	0.309	4.370(0.447–42.761)
**Coronary heart disease**	6(7.1)	4(3.4)	0.324	2.212(0.604–8.094)
**Atrial fibrillation**	25(29.8)	33(27.7)	0.755	1.104(0.596–2.045)
**eGFR<60 ml/min/1.73 m**^**2**^	16(19.0)	12(10.1)	0.097	2.098(0.935–4.706)
**Current smoking**	13(15.5)	17(14.3)	0.843	1.099(0.502–2.404)
**Alcohol consumption**	5(6.0)	10(8.4)	0.594	0.690(0.227–2.097)
**Previous MI history**	3(3.6)	3(2.5)	0.693	1.432(0.282–7.275)
**History of stroke**	11(13.1)	17(14.3)	0.840	0.904(0.400–2.044)
**Stroke severity on admission**				
**NIHSS score, median**	11.24(7.609)	9.54(6.955)	0.101	
**Antiplatelet therapy**	74(88.1)	106(89.1)	0.826	0.908(0.378–2.180)
**Anticoagulation therapy**	21(25.0)	45(37.8)	0.068	0.548(0.296–1.016)
**Thrombolysis therapy**	4(4.8)	1(0.8)	0.162	5.90(0.647–53.762)

Values are n (%) or mean ± SD.

*Abbreviations*: eGFR, estimulated glomerular filtration rate; MI, myocardial infarction.

Of the final set of 84 AIS patients with RHD (31 males; mean age, 61.6±12.2years), 49 (58.3%) were assigned to the elevated hs-cTnT group (≥ 14 ng/L; mean value, 55.3 ng/L; interquartile range, 3.7–1461 ng/L). Baseline characteristics of patients in the elevated hs-cTnT group(≥14ng/l) and the normal hs-cTnT group(<14ng/L) were compared (Table 2).At baseline, patients with elevated hs-cTnT levels showed significantly higher prevalence of hypertension and renal impairment than patients with normal hs-cTnT levels(both *P* = 0.024). Factors associated with elevated hs-cTnT in univariate analyses (*P*< 0.10) were entered into multivariate logistic regression, which identified only renal impairment as independently associated with hs-cTnT elevation(OR 4.184,95%CI 1.17 to 15.01,*P* = 0.028).

**Table 2:** Comparison of baseline characteristics between patients with elevated or normal hs-cTnT levels.

Variable	Elevated hs-cTnT(*n* = 49)	Normal hs-cTnT(*n* = 3)	*P*	OR (95%CI)
**Age, yr**	62.8(12.8)	59.8(11.42)	0.285	
**Female**	27(55.1%)	26(74.3%)	0.108	2.354(0.916–6.051)
**Time to admission, h**	72.4(115.3)	67.0(135.62)	0.845	
**Risk factors**				
**Hypertension**	16(32.7%)	4(11.4%)	0.024*	**3.758(1.131–12.48)**
**Diabetes mellitus**	2(4.1%)	0	0.226	1.043(0.984–1.105)
**Hyperlipidemia**	2(4.1%)	0	0.226	1.043(0.984–1.105)
**Seizure**	3(6.1%)	1(2.9%)	0.488	2.217(0.221–22.25)
**Coronary heart disease**	5(10.2%)	1(2.9%)	0.197	3.864(0.431–34.63)
**Atrial fibrillation**	17(34.7%)	8(22.9%)	0.242	1.793(0.67–4.797)
**eGFR<60 ml/min/1.73 m**^**2**^	16(32.7%)	4(11.4%)	0.024*	**3.758(1.131–12.48)**
**Current smoking**	8(16.3%)	5(14.3%)	0.799	1.171(0.348–3.936)
**Alcohol consumption**	3(6.1%)	2(5.7%)	0.938	1.076(0.17–6.804)
**Previous MI history**	3(6.1%)	0	0.136	1.065(0.992–1.144)
**History of stroke**	5(10.2%)	6(11.7%)	0.353	0.549(0.153–1.968)
**Stroke severity on admission**				
**NIHSS score, median**	12.0(8.0)	10.1(7.0)	0.255	
**PT on admission, sec**	13.2(3.9)	10.1(7.0)	0.265	
**APTT on admission, sec**	27.0(6.10)	25.8(6.90)	0.410	
**INR on admission**	1.21(0.37)	1.11(0.25)	0.157	
**Antiplatelet therapy**	42(85.7)	32(91.4)	0.511	0.563(0.135–2.347)
**Anticoagulation therapy**	10(20.4)	11(31.4)	0.310	0.559(0.207–1.515)
**Thrombolysis therapy**	2(4.1)	2(5.7)	0.729	0.702(0.094–5.240)

Values are n (%) or mean ± SD.

*Abbreviations*: APTT, activated partial thromboplastin time; eGFR, estimated glomerular filtration rate; INR, international standard ratio; PT, prothrombin time; MI, myocardial infarction.

### Hemorrhagic transformation (HT)

Of the 84 patients, 36 (42.9%) developed HT during the 1-year follow-up, and prevalence of HT was significantly higher among those with elevated hs-cTnT levels within 48 h after admission (55.1 vs25.7%, *P* = 0.007) (Table 3).After adjusting for age, sex, hypertension, renal impairment and NIHSS score on admission, risk of HT in patients with elevated hs-cTnT levels was 4.0-fold higher (95%CI 1.391 to 11.625,*P* = 0.01) than in patients with normal hs-cTnT levels (Table 4).

**Table 3:** Univariate analysis of hemorrhagic transformation(HT) or prognosis in patients with elevated or normal hs-cTnT levels.

Variable	Elevated hs-cTnT(*n* = 49)	Normal hs-cTnT(*n* = 35)	*P*	OR (95%CI)
**HT**	27/49(55.2)	9/35(25.7)	0.007*	**3.545(1.379–9.114)**
**Prognosis**				
**Death**				
**3 mos.**	14/49(28.6)	2/35(5.7)	0.009*	0.152(0.032–0.718)
**6 mos.**	15/49(30.6)	5/33(15.2)	0.110	0.405(0.131–1.252)
**12 mos.**	16/48 (33.3)	6/32(18.8)	0.152	0.462(0.158–1.348)
**Disability**				
**3 mos.**	16/35(45.7)	11/33(33.3)	0.297	1.684(0.630–4.501)
**6 mos.**	10/34(29.4)	6/28(21.4)	0.475	1.528(0.476–4.902)
**12 mos.**	7/32(21.9)	4/26(15.4)	0.531	1.540(0.397–5.973)
**Death/disability**				
**3 mos.**	30/49(61.2)	13/35(37.1)	0.029*	2.672(1.092–6.537)
**6 mos.**	25/49(51.0)	11/33(33.3)	0.113	2.083(0.834–5.203)
**12 mos.**	23/48(47.9)	10/32(31.3)	0.138	2.024(0.792–5.170)

**Table 4:** Multivariate analysis of hemorrhagic transformation (HT) and prognosis in patients with elevated or normal hs-cTnT levels.*

	P	OR	95%CI
**HT**	**0.010**	**4.021**	**1.391–11.625**
**Prognosis**			
**Death**			
**3 mos.**	**0.042**	**5.498**	**1.062–28.448**
**6 mos.**	0.292	2.058	0.630–6.727
**12 mos.**	0.311	1.806	0.578–5.639
**Disability**			
**3 mos.**	0.700	0.815	0.287–2.313
**6 mos.**	0.901	0.926	0.272–3.147
**12 mos.**	0.961	1.035	0.257–4.172
**Death/disability**			
**3 mos.**	0.154	2.230	0.740–6.720
**6 mos.**	0.466	1.691	0.412–6.940
**12 mos.**	0.380	2.080	0.405–10.676

*Data were adjusted for age, sex, hypertension, renal impairment and NIHSS score on admission.

### Prognosis

Mortality rates in the elevated hs-cTnT group were 28.6% at 3 months, 30.6% at 6 months, and 33.3% at 1 year (Table 3). The 3-month rate was significantly higher than that in the normal hs-cTnT group (*P* = 0.009).After adjusting for age, sex, hypertension, renal impairment, and NIHSS score on admission,3-month mortality in the elevated hs-cTnT group was 5.5-fold higher (95%CI1.062 to 28.448,*P* = 0.042) than in the normal hs-cTnT group (Table 4).The two groups did not, however, differ significantly in mortality rate at 6 months (*P* = 0.292) or 1 year (*P* = 0.311).

Rates of disability were similar between the two patient groups, though patients with elevated hs-cTnT levels showed a tendency toward higher rates at 3 months (45.7 vs 33.3%), 6 months (29.4 vs 21.4%) and 1 year (21.9% vs 15.4%).The combined rate of death/disability was significantly higher for the elevated hs-cTnT group at 3 months (*P* = 0.029, Table 3). This difference became insignificant after adjusting for age, sex, hypertension, renal impairment, and NIHSS score on admission (*P* = 0.154, Table 4). The rate of death/disability was similar between the two groups at 6 and 12 months (Table 4).

### Stroke recurrence

Nearly all patients were followed up for an entire year after stroke: only 4 of 84 patients (4.8%) were included in 3-month follow-up but lost to 1-year follow-up. By 1 year, 7 patients experienced stroke recurrence, which was diagnosed as ischemic stroke in all cases. Recurrence tended to be more prevalent among patients with elevated hs-cTnT levels than among those with normal levels (10.4% vs 6.3%, *P* = 0.518).

## Discussion

Here we provide preliminary evidence in a small cohort of Chinese patients with AIS and RHD that elevated hs-cTnT levels are associated with significantly greater risk of HT and 3-month mortality. The prevalence of elevated hs-cTnT levels in our cohort, defined as ≥14 ng/L, was 58.3%, consistent with previous studies[19,27]. Stroke may increase hs-cTnT levels through two mechanisms: when stroke coincides with acute coronary syndrome, causing ischemic myocardial necrosis[28]; or when stroke affects cortical areas controlling autonomic function, such as the insular cortex, causing autonomic imbalance and neurogenic cardiac damage[5,29].

In previous studies, it was found that cTnT levels did not increase greater than normal limits in patients with RHD because RHD [30] has a less destructive effect on the myocyte. However, in the current study, serum hs-cTnT levels in AIS patients with RHD increased much higher than that in other studies[19,27]. This might be explained by the fact that RHD and acute ischemic stroke interact with each other and strengthen the influence of rheumatic inflammatory process posing the damage to myocardial cells. The cause of injury may be the spread of inflammation to the adjacent myocardial tissue, which can eventually increase the serum hs-cTnT levels. Considering this, investigating the relationship and the mechanism between increased hs-cTnT levels and AIS patients with RHD will be the subject of further investigation.

We found that renal impairment was independently associated with elevated hs-cTnT levels within 48 h after admission, as in other studies[19,31], and that these elevated levels predicted HT after stroke. HT incidence was 42.9%, which falls within the broad incidence of 0.6–85% reported in a recent systematic review of stroke patients[32]. Since renal impairment by itself has already been shown to increase risk of spontaneous HT in stroke patients[33], it may be involved in the observed link between elevated hs-cTnT levels and HT after stroke. Controlling for renal impairment, as well as other potential confounders, in multivariate analysis showed that elevated hs-cTnT levels remained a significant predictor of HT, but further studies are needed to confirm that hs-cTnT levels on their own can contribute to HT.

Elevated hs-cTnT levels may cause HT through their association with hypercholesterolemia and arterial atherosclerosis[18], which can weaken the smooth muscle cells in arteries, influence vessel function and cause hemorrhage. Future work should examine this potential mechanism as well as others that may explain the observed association between high hs-cTnT levels and HT.

Our finding that elevated hs-cTnT levels were associated with mortality at 3 months, but not with mortality or other outcomes at 6 or 12 months, is consistent with previous studies.[31,34]It seems likely that changes in hs-cTnT levels over time, rather than a single hs-cTnT level, may predict long-term prognosis after stroke. Future studies should measure hs-cTnT levels over longer follow-up to explore fully the possible association between post-stroke evolution of hs-cTnT levels and long-term outcomes. Such monitoring may help identify individuals in need of further diagnostic testing or at higher risk of cardiac complications.

The results of this study should be interpreted carefully in light of its limitations. This was a single hospital-based study involving a small, highly specific patient population, so large cohorts, preferably from different ethnic groups and multiple medical centers, need to be studied. The statistical power of our study was limited by the fact that fewer than half of otherwise eligible patients could be included for lack of hs-cTnT measurements within 48 h after admission. This is because measurements were left up to the discretion of the physician, so a prospective study in which such levels are measured systematically is needed. In such a study, hs-cTnT levels should be measured repeatedly to allow longitudinal analysis. Further work should also examine whether the relationship between hs-cTnT levels and HT is relevant to other types of stroke patients and to the general population, as well as whether post-stroke levels of hs-cTnT differ between patients with and without RHD.

## Conclusions

More than half of stroke patients with RHD have elevated hs-cTnT levels, which are associated with higher risk of HT and 3-month mortality. These preliminary results should be verified and extended in future studies with large cohorts.

## Supporting Information

S1 File File containing patient information. **Table A:** Baseline characteristics of Chinese acute ischemic stroke patients with Rheumatic Heart Disease from the Chengdu Stroke Registry who were included or excluded from the present study. **Table B:** Comparison of baseline characteristics between patients with elevated or normal hs-cTnT levels. **Table C:** Univariate analysis of hemorrhagic transformation (HT) or prognosis in patients with elevated or normal hs-cTnT levels. **Table D:** Multivariate analysis of hemorrhagic transformation (HT) and prognosis in patients with elevated or normal hs-cTnT levels.
(DOCX)
