# Supplementary material for: Association of Elevated High Sensitivity Cardiac Troponin T(hs-cTnT) Levels with Hemorrhagic Transformation and 3-Month Mortality in Acute Ischemic Stroke Patients with Rheumatic Heart Disease in China
Source: PLoS One. 2016 Feb 5;11(2):e0148444. doi: 10.1371/journal.pone.0148444 (PMC4744030; doi:10.1371/journal.pone.0148444)
Supplement: S1 File — Table A: Baseline characteristics of Chinese acute ischemic stroke patients with Rheumatic Heart Disease from the Chengdu Stroke Registry who were included or excluded from the present study. Table B: Comparison of baseline characteristics between patients with elevated or normal hs-cTnT levels. Table C: Univariate analysis of hemorrhagic transformation (HT) or prognosis in patients with elevated or normal hs-cTnT levels. Table D: Multivariate analysis of hemorrhagic transformation (HT) and prognosis in patients with elevated or normal hs-cTnT levels. (DOCX) [file pone.0148444.s001.docx]

**Table A. Baseline characteristics of Chinese acute ischemic stroke patients with Rheumatic Heart Disease from the Chengdu Stroke Registry who were included or excluded from the present study.**

| Variable | Included  (*n*=84) | Excluded  (*n*=119) | *P* | | | OR (95%CI) |
| --- | --- | --- | --- | --- | --- | --- |
| Age,yr | 61.58(12.24) | 58.79(12.7) | | 0.119 |  | |
| Female | 53(63.1) | 78(65.5) | | 0.951 | 1.019(0.568-1.826) | |
| Time to admission, h | 70.11(123.34) | 70.24(91.53) | | 0.993 |  | |
| Risk factors |  |  | |  |  | |
| Hypertension | 20(23.8) | 23(19.3) | | 0.487 | 1.304(0.662-2.568) | |
| Diabetes mellitus | 2(2.4) | 8(6.7) | | 0.201 | 0.338(0.070-1.636) | |
| Hyperlipidemia | 2(2.4) | 3(2.5) | | 0.949 | 0.943(0.154-5.771) | |
| Seizure | 3(3.6) | 1(0.8) | | 0.309 | 4.370(0.447-42.761) | |
| Coronary heart disease | 6(7.1) | 4(3.4) | | 0.324 | 2.212(0.604-8.094) | |
| Atrial fibrillation | 25(29.8) | 33(27.7) | | 0.755 | 1.104(0.596-2.045) | |
| eGFR<60 ml/min/1.73 m^2^ | 16(19.0) | 12(10.1) | | 0.097 | 2.098(0.935-4.706) | |
| Current smoking | 13(15.5) | 17(14.3) | | 0.843 | 1.099(0.502-2.404) | |
| Alcohol consumption | 5(6.0) | 10(8.4) | | 0.594 | 0.690(0.227-2.097) | |
| Previous MI history | 3(3.6) | 3(2.5) | | 0.693 | 1.432(0.282-7.275) | |
| History of stroke | 11(13.1) | 17(14.3) | | 0.840 | 0.904(0.400-2.044) | |
| Stroke severity on admission |  |  | |  |  | |
| NIHSS score, median | 11.24(7.609) | 9.54(6.955) | | 0.101 |  | |
| Antiplatelet therapy | 74(88.1) | 106(89.1) | | 0.826 | 0.908(0.378-2.180) | |
| Anticoagulation therapy | 21(25.0) | 45(37.8) | | 0.068 | 0.548(0.296-1.016) | |
| Thrombolysis therapy | 4(4.8) | 1(0.8) | | 0.162 | 5.90(0.647-53.762) | |

Values are n (%) or mean ± SD

*Abbreviations:* eGFR, estimulated glomerular filtration rate; MI, myocardial infarction

**Table B. Comparison of baseline characteristics between patients with elevated or normal hs-cTnT levels**

| Variable | Elevated hs-cTnT  (*n*=49) | Normal hs-cTnT  (*n*=35) | *P* | | | OR (95%CI) |
| --- | --- | --- | --- | --- | --- | --- |
| Age, yr | 62.8(12.8) | 59.8(11.42) | | 0.285 |  | |
| Female | 27(55.1%) | 26(74.3%) | | 0.108 | 2.354(0.916-6.051) | |
| Time to admission, h | 72.4(115.3) | 67.0(135.62) | | 0.845 |  | |
| Risk factors |  |  | |  |  | |
| Hypertension | 16(32.7%) | 4(11.4%) | | **0.024*** | **3.758(1.131-12.48)** | |
| Diabetes mellitus | 2(4.1%) | 0 | | 0.226 | 1.043(0.984-1.105) | |
| Hyperlipidemia | 2(4.1%) | 0 | | 0.226 | 1.043(0.984-1.105) | |
| Seizure | 3(6.1%) | 1(2.9%) | | 0.488 | 2.217(0.221-22.25) | |
| Coronary heart disease | 5(10.2%) | 1(2.9%) | | 0.197 | 3.864(0.431-34.63) | |
| Atrial fibrillation | 17(34.7%) | 8(22.9%) | | 0.242 | 1.793(0.67-4.797) | |
| eGFR<60 ml/min/1.73 m^2^ | 16(32.7%) | 4(11.4%) | | **0.024*** | **3.758(1.131-12.48)** | |
| Current smoking | 8(16.3%) | 5(14.3%) | | 0.799 | 1.171(0.348-3.936) | |
| Alcohol consumption | 3(6.1%) | 2(5.7%) | | 0.938 | 1.076(0.17-6.804) | |
| Previous MI history | 3(6.1%) | 0 | | 0.136 | 1.065(0.992-1.144) | |
| History of stroke | 5(10.2%) | 6(11.7%) | | 0.353 | 0.549(0.153-1.968) | |
| Stroke severity on admission |  |  | |  |  | |
| NIHSS score, median | 12.0(8.0) | 10.1(7.0) | | 0.255 |  | |
| PT on admission, sec | 13.2(3.9) | 10.1(7.0) | | 0.265 |  | |
| APTT on admission, sec | 27.0(6.10) | 25.8(6.90) | | 0.410 |  | |
| INR on admission | 1.21(0.37) | 1.11(0.25) | | 0.157 |  | |
| Antiplatelet therapy | 42(85.7) | 32(91.4) | | 0.511 | 0.563(0.135-2.347) | |
| Anticoagulation therapy | 10(20.4) | 11(31.4) | | 0.310 | 0.559(0.207-1.515) | |
| Thrombolysis therapy | 2(4.1) | 2(5.7) | | 0.729 | 0.702(0.094-5.240) | |

Values are n (%) or mean ± SD

*Abbreviations:* APTT, activated partial thromboplastin time; eGFR, estimated glomerular filtration rate; INR, international standard ratio; PT, prothrombin time; MI, myocardial infarction

**Table C Univariate analysis of hemorrhagic transformation(HT) or prognosis in patients with elevated or normal hs-cTnT levels**

| Variable | Elevated hs-cTnT  (*n*=49) | Normal hs-cTnT  (*n*=35) | *P* | OR (95%CI) |
| --- | --- | --- | --- | --- |
| HT | 27/49(55.2) | 9/35(25.7) | **0.007*** | **3.545(1.379-9.114)** |
| Prognosis |  |  |  |  |
| Death |  |  |  |  |
| 3 mos. | 14/49(28.6) | 2/35(5.7) | **0.009*** | **0.152(0.032-0.718)** |
| 6 mos. | 15/49(30.6) | 5/33(15.2) | 0.110 | 0.405(0.131-1.252) |
| 12 mos. | 16/48（33.3） | 6/32(18.8) | 0.152 | 0.462(0.158-1.348) |
| Disability |  |  |  |  |
| 3 mos. | 16/35(45.7) | 11/33(33.3) | 0.297 | 1.684(0.630-4.501) |
| 6 mos. | 10/34(29.4) | 6/28(21.4) | 0.475 | 1.528(0.476-4.902) |
| 12 mos. | 7/32(21.9) | 4/26(15.4) | 0.531 | 1.540(0.397-5.973) |
| Death/disability |  |  |  |  |
| 3 mos. | 30/49(61.2) | 13/35(37.1) | **0.029*** | **2.672(1.092-6.537)** |
| 6 mos. | 25/49(51.0) | 11/33(33.3) | 0.113 | 2.083(0.834-5.203) |
| 12 mos. | 23/48(47.9) | 10/32(31.3) | 0.138 | 2.024(0.792-5.170) |

**Table D. Multivariate analysis of hemorrhagic transformation (HT) and prognosis in patients with elevated or normal hs-cTnT levels***

|  | P | OR | 95%CI |
| --- | --- | --- | --- |
| HT | **0.010** | **4.021** | **1.391-11.625** |
| Prognosis |  |  |  |
| Death |  |  |  |
| 3 mos. | **0.042** | **5.498** | **1.062–28.448** |
| 6 mos. | 0.292 | 2.058 | 0.630-6.727 |
| 12 mos. | 0.311 | 1.806 | 0.578-5.639 |
| Disability |  |  |  |
| 3 mos. | 0.700 | 0.815 | 0.287-2.313 |
| 6 mos. | 0.901 | 0.926 | 0.272-3.147 |
| 12 mos. | 0.961 | 1.035 | 0.257-4.172 |
| Death/disability |  |  |  |
| 3 mos. | 0.154 | 2.230 | 0.740-6.720 |
| 6 mos. | 0.466 | 1.691 | 0.412-6.940 |
| 12 mos. | 0.380 | 2.080 | 0.405-10.676 |

*****Data were adjusted for age, sex, hypertension, renal impairment and NIHSS score on admission.
